# Transcriptome sequencing and differential expression analysis of seed starch accumulation in Chinese chestnut Metaxenia

**DOI:** 10.1186/s12864-021-07923-5

**Published:** 2021-08-13

**Authors:** Shengxing Li, Zhuogong Shi, Qiurong Zhu, Liang Tao, Wenhui Liang, Zhiheng Zhao

**Affiliations:** Guanxi forestry research institute, Nanning, Guanxi China

**Keywords:** Chestnut, Metaxenia, Differential gene expression, Seed development, Starch synthesis

## Abstract

**Background:**

Chestnut seeds are important kinds of edible nuts rich in starch and protein. The characteristics and nutrient contents of chestnut have been found to show obvious metaxenia effects in previous studies. To improve the understanding of the effect of metaxenia on chestnut starch and sucrose metabolism, this study used three varieties of chestnut, ‘Yongfeng 1’, ‘YongRen Zao’ and ‘Yimen 1’, as male parents to pollinate the female parent, ‘Yongfeng 1’, and investigated the mechanisms of starch and sucrose metabolism in three starch accumulation stages (70 (S1), 82 (S2), and 94 (S3) days after pollination, DAP) in chestnut seed kernels.

**Result:**

Most carbohydrate metabolism genes were highly expressed in YFF (self-pollinated ‘Yongfeng 1’) in stage S2 and in YFR (‘Yongfeng 1’ × ‘Yongren Zao’) and YFM (‘Yongfeng 1’ × ‘Yimen 1’) in stage S3. In stage S3, hub genes encoding HSF_DNA-binding, ACT, Pkinase, and LIM proteins and four transcription factors were highly expressed, with YFF showing the highest expression, followed by YFR and YFM. In addition, transcriptome analysis of the kernels at 70, 82 and 94 DAP showed that the starch granule-bound starch synthase (EC 2.4.1.242) and ADP-glucose pyrophosphorylase (EC 2.7 .7.27) genes were actively expressed at 94 DAF. Chestnut seeds regulate the accumulation of soluble sugars, reducing sugars and starch by controlling glycosyl transferase and hydrolysis activity during development.

**Conclusion:**

These results and resources have important guiding significance for further research on starch and sucrose metabolism and other types of metabolism related to chestnut metaxenia.

**Supplementary Information:**

The online version contains supplementary material available at 10.1186/s12864-021-07923-5.

## Background

Chinese chestnut (*C. mollissima* BL.) has been cultivated for its nuts and timber for over 4000 years [1]. Over 300 cultivars have been selected for nut production [2]. It remains a very important commercial crop in China. As of 2018, Chinese chestnut was widely grown in Asia, Europe, and America for nut production, accounting for more than 80% of the global production (adopted from the UN Food and Agriculture Organization, Corporate Statistical Database: http://www.fao.org/). Chinese chestnut is a rich source of vitamin C, protein, and a variety of minerals, with particularly high contents of magnesium, phosphorus, and potassium (adopted from USDA FoodData Central: https://fdc.nal.usda.gov/). However, Chinese chestnut cultivation continues to face serious problems, such as uneven quality, the production of many hollow bracts and low yields [3–5]. Chinese chestnut shows typical cross-pollination characteristics and a low self-pollination seed setting rate; therefore, it is very important to separate pollinated trees to improve chestnut yields [6, 7].

The phenomenon in which the endosperm of a modern hybrid seed displays paternal genetic traits is known as xenia [8]. Xenia has applications not only in genetic and physiological research but also in plant breeding and crop production [9]. Xenia includes metaxenia, which is the effect of pollen on fruit size and other fruit characteristics [10, 11]. Metaxenia has been described in many species, such as apples [12], maize [13], and blueberries [14]. Previous studies have shown that the traits and composition of chestnut fruits exhibit obvious metaxenia effects [15–17]. In addition, pollination treatments result in substantial differences in chestnut ovary nutrition [18]. Therefore, studying the metaxenia effect is very important for the reasonable allocation of chestnut trees used for pollination, for the scientific planning of new chestnut gardens and for improving the quality of chestnut fruit.

Chestnut seed starch accumulation is a very complex process involving many expression-dependent physiological changes and the regulation of a large number of genes and phytohormones. Chinese chestnut flowers in mid-summer, and it takes approximately 100 days for its nuts to fully ripen [19]. Starch is the major metabolite in chestnuts [20], and the starch accumulation period of chestnut seeds is the month before maturation. Zhang et al. [21] compared gene expression profiles between two stages (45 vs 75 DAF, days after flowing) of Chinese chestnut and identified two granule-bound starch synthase unigenes exhibiting two fold higher expression at 75 DAF than at 45 DAF. Two starch branching enzyme isoforms of Chinese chestnut were identified through zymogram analysis. The gene expression of these two CmSBE isoforms increased beginning 64 days after pollination (DAP) and reached the highest levels at 77 DAP [22]. Transcriptomic profiling of Chinese chestnut kernels at 70, 82, and 95 DAF indicated that soluble starch synthase and α-1,4-glucan branching enzyme genes were actively expressed at 82 and 94 DAF. In addition, starch and sucrose metabolism was significantly enriched in all comparisons included in the study [19].

Although the regulatory networks of chestnut seed development have been studied, there are few reports on the gene regulatory network governing seed metaxenia during the maturation of chestnuts supported by chestnut genome analysis. The first whole-genome sequence of *C. mollissima*, which was completed in 2019 [23], provides a promising resource for chestnut functional genomic research. Here, we used ‘Yongfeng 1’, ‘Yongren Zao’ and ‘Yimen 1’ as male parents to pollinate ‘Yongfeng 1’ as the female parent, and we explored the molecular mechanism of the seed metaxenia effect on starch accumulation. We characterized seed development in chestnuts by evaluating nutrient accumulation, using ELISA to quantify enzyme activity, examining the expression of key genes through transcriptome profiling and verifying the expression of genes related to starch synthesis by qRT-PCR. By analyzing the correlations between nutrient changes and seed development, we determined the gene regulatory network controlling the accumulation of starch and soluble sugars in chestnut seed metaxenia and analyzed key genes. We provide an abundance of genomic resources for chestnuts and novel molecular insights into the correlations between physiological changes and starch accumulation in chestnut metaxenia.

## Results

### Physiological description of the starch accumulation period related to chestnut seed metaxenia

**Fig. 1:**
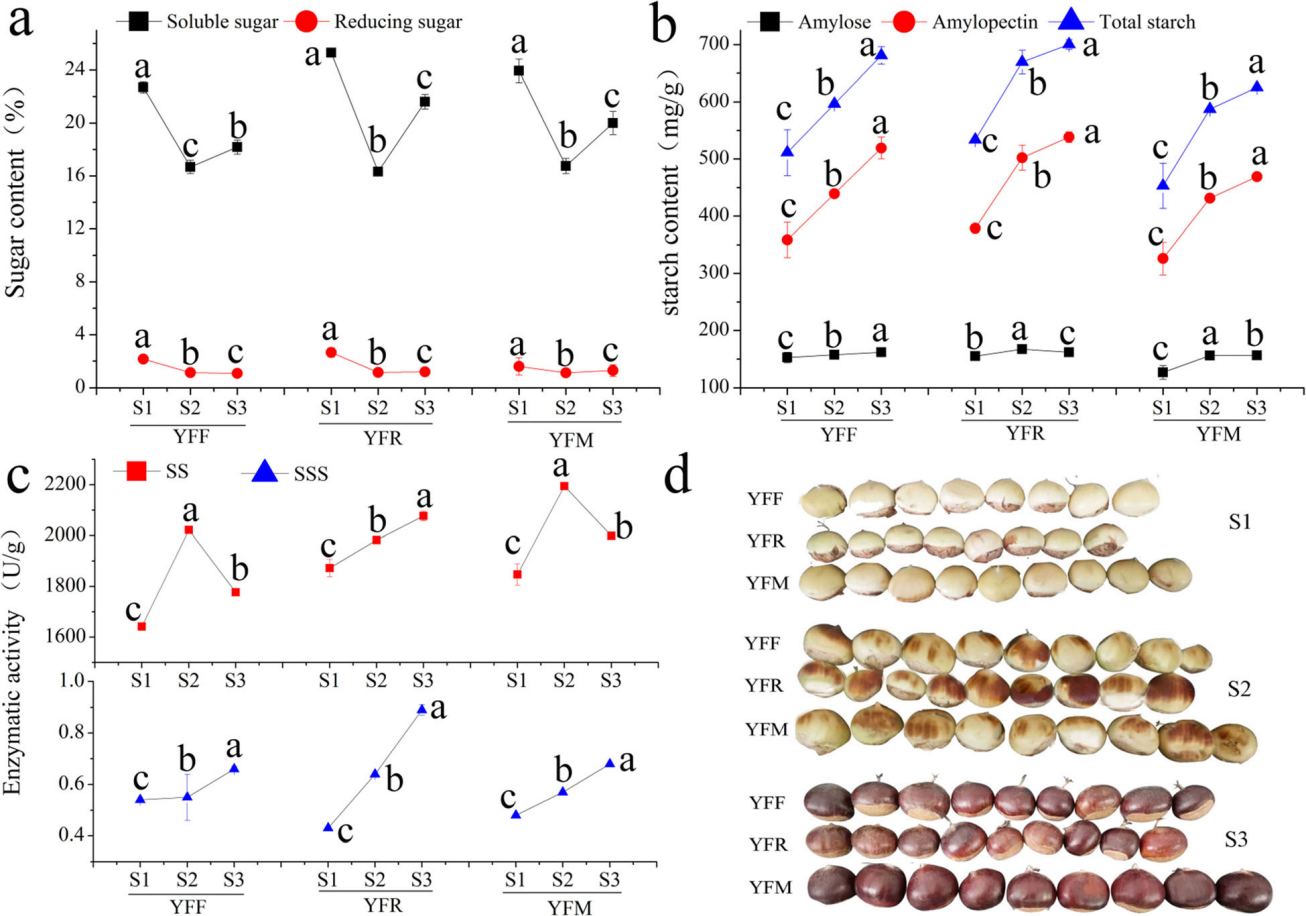
Chestnut samples and physiological characteristics at three stages. **A** Comparison of sugar contents in three stages. **B** Comparison of starch contents in three stages. **C** Comparison of enzyme activity in three stages. **D** Illustration of seeds at three stages that were included in the study. Note: Lowercase letters indicate significant differences (*P* < 0.05)

The process of chestnut seed starch accumulation can be divided into three stages (S1, S2 and S3) based on changes at the morphological and physiological levels. The seed collection used in this study was obtained from the following different pollination combinations: YFF, YFR and YFM. As shown in Fig. 1, the seed coloration of YFF, YFR and YFM was similar in the three developmental stages. However, there were significant differences in the seed sizes of YFF, YFR and YFM at different developmental stages. The seed colors of YFF, YFR and YFM at the S1 (70 DAP), S2 (82 DAP) and S3 (94 DAP) stages were yellow, yellow and brown, and brown, respectively. The seeds of YFF were smaller than those of YFR and YFM at all three stages, which indicated that metaxenia had a significant effect on the fruit size (Fig. 1d).

The sugar content, starch content, sucrose synthase (SS) activity and starch synthase (SSS) activity were evaluated in chestnut seeds at different developmental stages (Fig. 1a, b, c). There were significant differences in sugar content and SS activity in the seeds of YFF, YFR and YFM at different developmental stages, indicating that metaxenia played a vital role in chestnut seed maturation. The mean soluble sugar content peaked in the S1 stage, significantly decreased in stage S2 and slightly increased in stage S3 in chestnut seeds. Moreover, the sugar content of YFR was higher than those of YFM and YFF, and YFM exhibited higher SS activity than YFR and YFF, implying that the seeds of YFR ripened more quickly than those of YFM and YFF. Furthermore, the starch content and SSS activity in the seeds of YFF, YFR and YFM exhibited apparent differences, which also indicated that metaxenia played a vital role in chestnut seed maturation. The total starch content, amylose content and SSS activity in the seeds of YFF, YFR and YFM increased with the maturity of the seeds. Moreover, the starch content was related to the seed color—the darker the seed color was, the higher the starch content was. The sugar content and SS activity were also correlated with the starch content and SSS activity in chestnut seeds. The pollination combinations resulting in a higher sugar contents also resulted in higher starch contents, and vice versa. Taken together, the results indicated that the sugar and starch contents presented obvious metaxenia effects during the seed starch accumulation period, and the metaxenia effects obviously influenced SS activity and SSS activity.

### A global transcriptome comparison of starch accumulation at different stages

To explore the potential molecular mechanism of starch accumulation, global transcriptome analysis was performed on chestnut seeds at different developmental stages. RNA was isolated from seeds of YFF, YFR and YFM at S1, S2 and S3 (referred to as YFF-S1, YFF-S2, YFF-S3, YFR-S1, YFR-S2, YFR-S3, YFM-S1, YFM-S2 and YFM-S3), and 27 cDNA libraries (with three independent biological replicates for each sample) were constructed (Additional file 1: Figure S1). After large-scale sequencing, over 1.14 billion high-quality reads were generated. Approximately 45 million high-quality reads were acquired from each biological replication (Additional file 1: Table S1). The high-quality reads were mapped to the chestnut genome with the TopHat mapping tool. After treatment with Cufflinks and Cuffmerge, 21,423 genetic loci present in every sample were obtained. Notably, 9999 novel genes were detected. The high-quality reads that were uniquely mapped to the chestnut genome were used for quantification via the fragments per kilobase of transcript length per million mapped reads (FPKM) method. The Spearman correlation coefficients (SCCs) of the biological replicates for each sample ranged from 0.785 to 0.94, except for one replicate each of YFF-S1 and YFR-S1, which were not used for the analysis. These results verified the quality of the obtained reads.

**Fig. 2:**
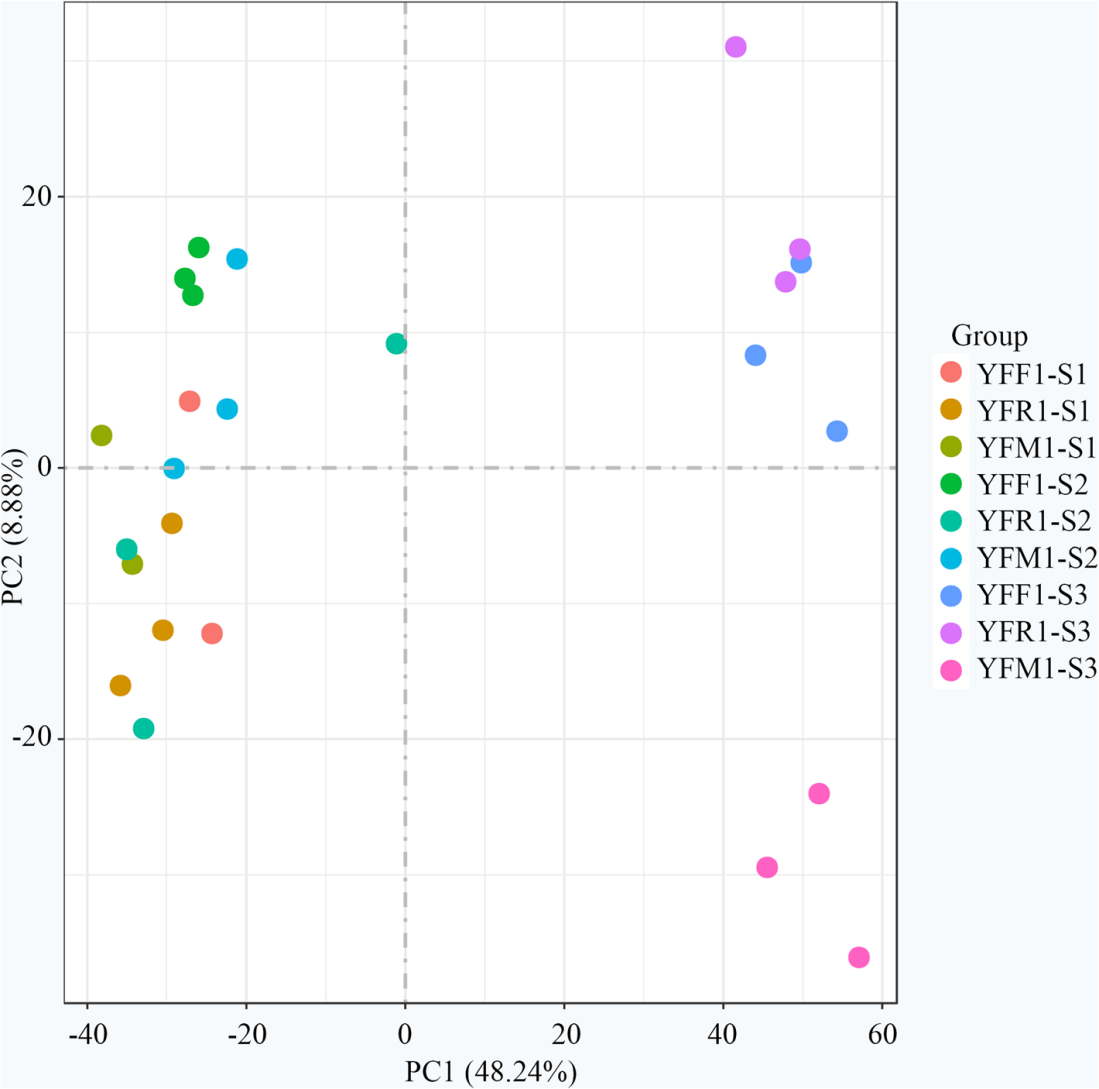
Principal component analysis (PCA) of the global transcriptome obtained from three pollination combinations

To explore the global differences in transcriptome dynamics during seed starch accumulation in YFF, YFR and YFM, principal component analysis (PCA) of the average FPKM values was performed based on the SCC analysis (Fig. 2, Additional file 1: Figure S2a). The stages showing higher correlations might present more similar transcriptomes and functions. These analyses indicated that there was a higher correlation between similar developmental stages among YFF, YFR and YFM. As expected, the analysis yielded lower correlations during different developmental stages in YFF, YFR and YFM. Although there was a high correlation between stages S1 and S2, stage S3 showed a low correlation with stages S1 and S2. Interestingly, the clustering of YFF and YFR was obviously different. At stage S3, the clustering of YFM was obviously different from that of YFF, which presented a closer correlation with YFR. These results indicated that the seeds of YFR developed faster than those of YFF in the early stages of seed development. The seed development of YFM was similar to that of YFR at the early stage and slower than that of YFR at the later period, which was consistent with the seed morphology of the two chestnut lines. Taken together, these results indicated that there were major differences in transcription in the three stages of the starch accumulation period in the seeds of each pollination combination.

### Differentially expressed genes during the chestnut seed starch accumulation period

**Fig. 3:**
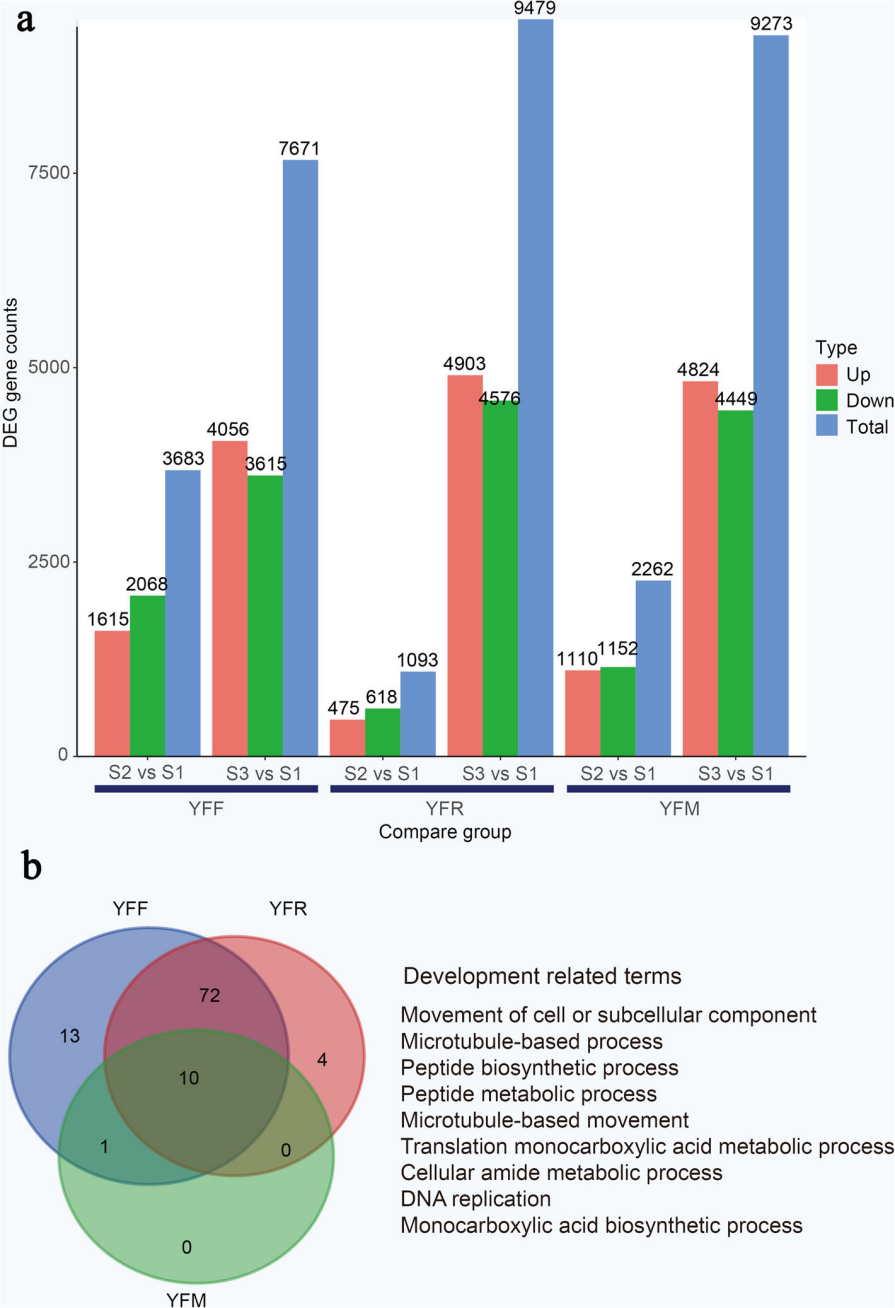
DEG counts and the top eight GO terms in seeds from three pollination combinations. **A** Comparison of DEGs in seeds from different pollination combinations. **B** Development-related GO terms in seeds from three pollination combinations

To better understand gene expression dynamics during the chestnut seed starch accumulation period, differentially expressed genes (DEGs) between these nine samples were identified using the BH algorithm. The differential expression analysis revealed a large number of candidate DEGs according to the following criteria: DEGs were defined according to the ratio of FPKM expression values, a false discovery rate (FDR) < 0.05 and an absolute |log_2_ (fold-change) | value > 0. The genes that were specifically expressed at each stage of the starch accumulation period in the seeds from these three pollination combinations were identified (Fig. 3a). A Venn diagram showed that 1874, 487 and 700 genes were specifically expressed in stage S2 in YFF, YFR and YFM, respectively. In total, 5862, 8873 and 7711 genes were specifically expressed in stage S3 in YFF, YFR and YFM, respectively. Genes that were specifically expressed in different varieties were also identified (Additional file 1: Figure S2a). These results suggested that the highest chestnut seed starch accumulation activity occurred in the S3 stage. Moreover, the most significant starch accumulation was observed in YFR seeds in the S3 stage, which might lead to early maturity due to metaxenia.

The gene ontology (GO) analysis of DEGs between the seeds of YFF, YFR and YFM at different developmental stages. The results show many GO terms related to seed development and starch accumulation (Additional file 1: Figure S2b). These processes are relevant to seed development and starch accumulation. In addition, the GO enrichment analysis of DEGs showed that the S2 stage was characterized by terms related to the cell cycle and growth. The S3 stage was associated with terms related to cell walls, lipid metabolism, secondary metabolism, and protein synthesis. At stage S3, the four most significantly enriched GO terms were amide metabolic process, monocarboxylic acid metabolic process, organic acid metabolic process and protein biosynthesis. In addition, overlap analysis of GO terms that were significantly enriched in each variety showed that several terms were either specifically enriched in one variety or generally enriched in two varieties (Fig. 3b). For example, GO terms related to carbohydrate derivative metabolic, organophosphate biosynthetic, organophosphate metabolic, and cellular catabolic processes were unique to YFF, while those related to DNA metabolic and amino acid metabolic processes were unique to YFR. GO terms related to microtubule-based, peptide metabolic, translation, monocarboxylic acid metabolic, cellular amide metabolic processes and DNA replication were found in all three varieties. Taken together, these results suggested that certain groups of genes exhibited specific functions in specific stages of the chestnut starch accumulation period. The processes of energy metabolism, organic acid metabolism and translation were presumably activated to promote the synthesis of ATP so that the seeds could obtain more energy during this time. Additionally, ribosomes increase the capacity for protein (peptide) synthesis, and the gluconeogenic pathway enhances sugar accumulation in chestnut seeds. In conclusion, in the early stage of seed development, energy metabolism, organic acid metabolism and translation processes are highly activate, which promotes the synthesis of ATP so that the seeds can obtain more energy. The middle and late stages are characterized by the activation of starch and sugar metabolism, and the accumulation of carbohydrates in chestnut is promoted.

### Expression patterns of genes responsible for starch accumulation

**Fig. 4:**
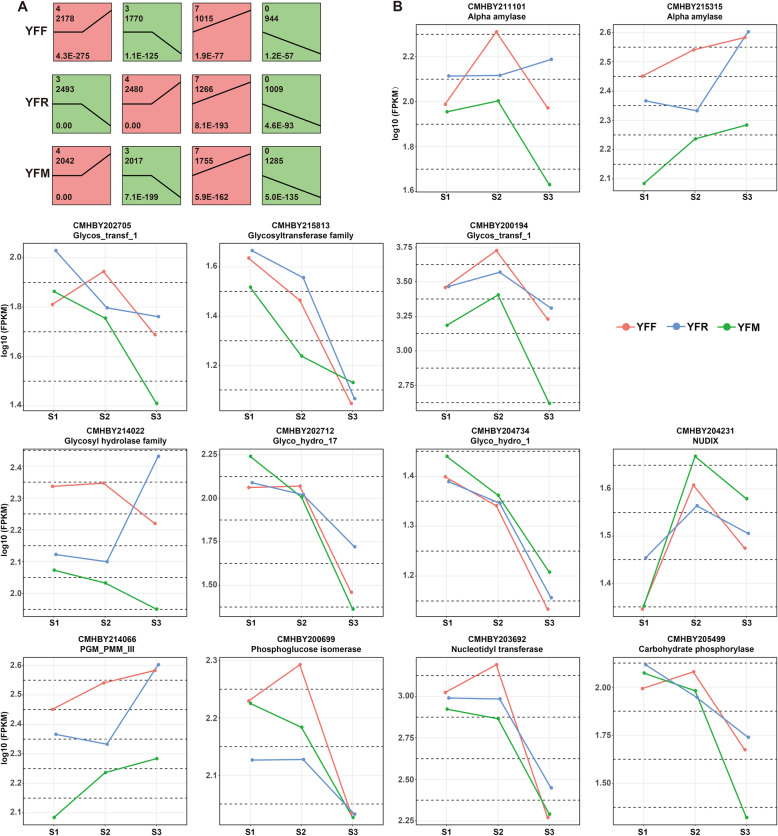
Expression patterns of genes in the seeds of three pollination combinations at three stages. **A** Expression patterns of all genes, **B** expression patterns of key genes

To analyze the expression profiles of the identified DEGs, 9545, 9966 and 9973 DEGs derived from YFF, YFR and YFM, respectively, were used for cluster analysis with Short Time-series Expression Miner (STEM). Specifically, 5892 DEGs from YFF and 3325 DEGs from YFM were significantly (*P* value≤0.05) aggregated into four profiles, including two downregulated modes and two upregulated modes, and the expression patterns of YFF and YFM were consistent. The 6737 DEGs from YFR were also significantly (*P* value≤0.05) aggregated into four profiles, including two downregulated modes and two upregulated modes, but the YFR expression pattern was inconsistent with those of YFF and YFM (Fig. 4a). GO analysis divided the genes in each profile into three major groups: biological process, molecular function and cellular component. Among the identified cellular components, a large number of upregulated and downregulated DEGs were related to cells, cell parts and organelles. Among biological processes, the two most enriched GO subcategories were cellular processes and metabolic processes. Similarly, in the molecular function category, the two most enriched GO subcategories were catalytic activity and binding.

### Profiles of transcription factors (TFs) involved in carbohydrate metabolism in seeds

The expression profiles of these genes were analyzed to explore the expression patterns of key genes encoding five TFs involved in carbohydrate metabolism in seeds. The five TFs were Glycos_transf_1, PGM_PMM_III, Glyco_hydro_17, NUDIX and Glyco_hydro_1. Most of these genes were highly expressed in YFF in stage S2 (Fig. 4b) and in YFR and YFM in stage S3. The difference in gene expression found in YFF versus YFR and YFM in the S3 stage was mainly due to the metaxenia effect. The expression of genes encoding glycosyl transferase, carbohydrate phosphorylase, phosphoglucose isomerase, NUDIX and nucleotidyl transferase in the seeds of YFF was increased in stage S2 compared to stage S1 and then decreased in stage S3, which was consistent with the variation trend of SS activity. In the seeds of YFR, the expression of genes encoding alpha amylase increased with seed maturity, which was consistent with the variation trend of the starch contents. However, the expression of genes encoding glycosyl hydrolase and phosphoglucomutase/phosphomannomutase decreased with seed maturity in the seeds of all three pollination combinations. These enzymes are responsible for the biosynthesis of intracellular sugars and participate in the production of energy. These results indicated that the synthesis of soluble sugars, reducing sugars and SS was controlled by regulating the transcriptional activity of the gene encoding glycosyl transferase. However, starch accumulation was related to the phosphorylation of glycosyl hydrolase and glucomutase.

### Hierarchical clustering of all DEGs

Differences in gene expression and regulatory networks are important to elucidate regulatory relationships in seed starch accumulation. To explore the gene regulation network during seed development, coexpressed gene sets of highly expressed genes were identified by weighted gene coexpression network analysis (WGCNA). As shown in the tree diagram (Additional file 1: Figure S3 a), we performed hierarchical clustering of 2345 genes, and in total, 22 different modules, each of which contained 85 to 2222 genes, were identified. Then, each coexpression module was correlated with the seed development stage by Pearson correlation coefficient analysis. Interestingly, three coexpression modules were uniquely associated with specific stages of seed development, among which the turquoise module, red module and blue module were related to stages S1, S2 and S3, respectively (Additional file 1: Figure S3 b). Moreover, many modules were associated with more than one seed development stage, and some of these modules were related only to the specific developmental stage of a particular variety. For example, the royal blue module and salmon module were highly correlated with stage S3 in YFF and YFM, respectively.

**Fig. 5:**
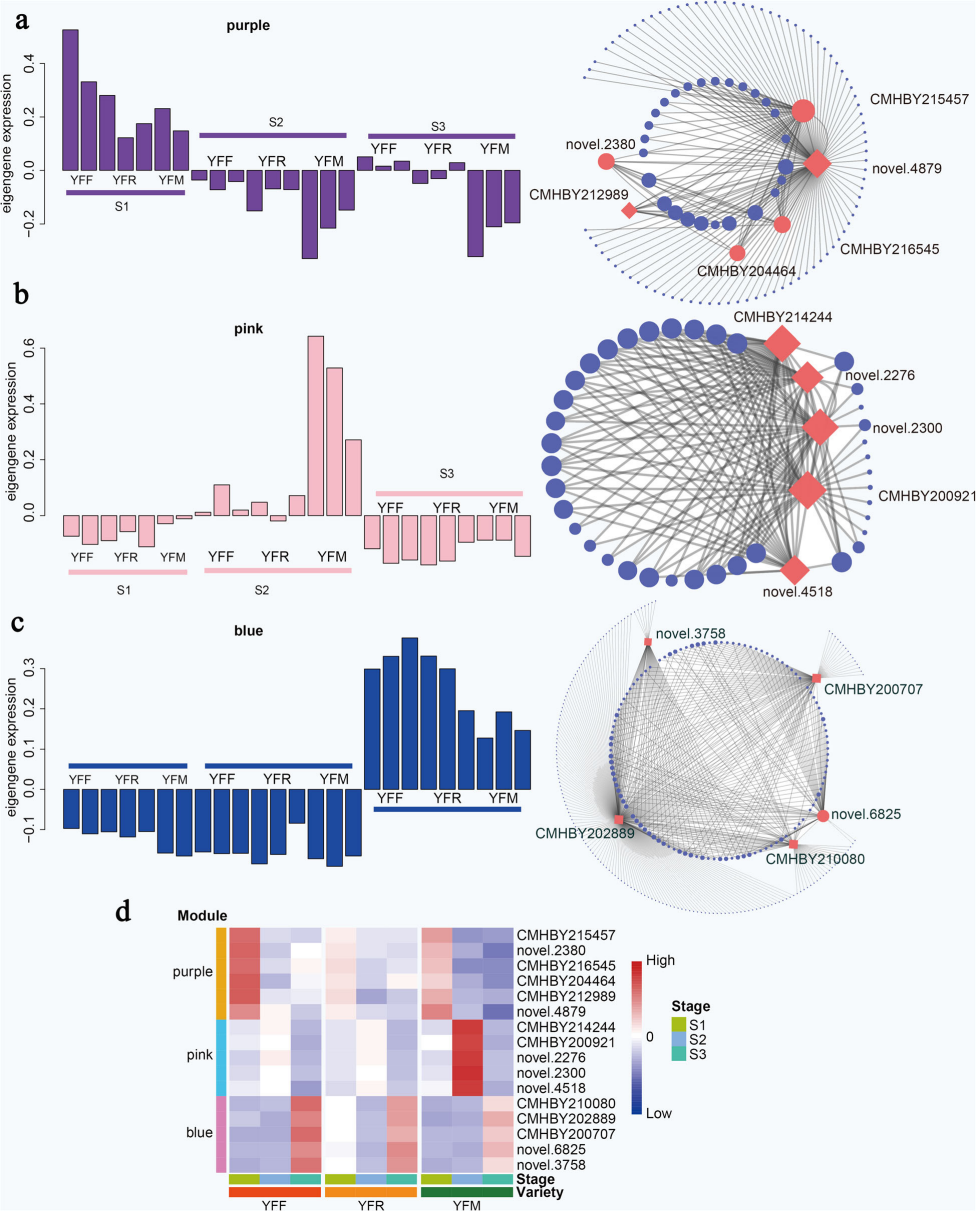
Analysis of key modules based on WGCNA. **A** Purple module key genes; **B** pink module key genes; **C** blue module key genes; **D** expression patterns of TFs

Hub genes related to chestnut seed starch accumulation in different stages were identified by WGCNA (Fig. 5a, b, c). The genes in the purple module were expressed at the highest levels in the early stages of seed development (S1). The genes in the pink module exhibited the highest level of expression in stage S2. The genes in the blue module were expressed at the highest levels in stage S3. In the purple module, six hub genes related to protein translation (ribosomal protein and RNA recognition), cell proliferation and sugar biosynthesis were confirmed. Five hub genes involved in translocation, one hub gene encoding transmethylase and one hub gene related to promoting cell proliferation were identified in the pink module. Considering the crucial role of TFs in gene expression and plant development, the expression patterns of TFs were analyzed (Fig. 5d). The RRM and Ras family TFs among these hub genes that were closely related to cell proliferation exhibited particularly high transcriptional activity at stage S1. ABC transporter (novel.2300 and novel.2276) and sulfate transporter (CMHBY214244) family genes were highly expressed in stage S2, especially in YFM, suggesting that a large number of genes related to substance transport in seeds were highly expressed under the regulation of TFs. Taken together, the results indicated that large amounts of materials and energy were transported to seeds for anabolic reactions, which was one of the reasons for observing the highest sugar content in seeds in stage S2.

In the blue module, we identified a set of hub genes encoding HSF_DNA-binding, ACT, Pkinase, and LIM proteins and four TFs that were highly expressed in stage S3. The expression of these genes was highest in YFF, followed by YFR and YFM. The differences in the expression of these genes between the seeds of different pollination combinations were mainly due to the metaxenia effect. These genes play important roles in plant growth, hormone responses and abiotic and biotic stress responses. They might participate in regulating cell senescence and death in chestnut seeds.

### Analysis of gene expression related to sucrose and starch metabolism in chestnut seeds

**Fig. 6:**
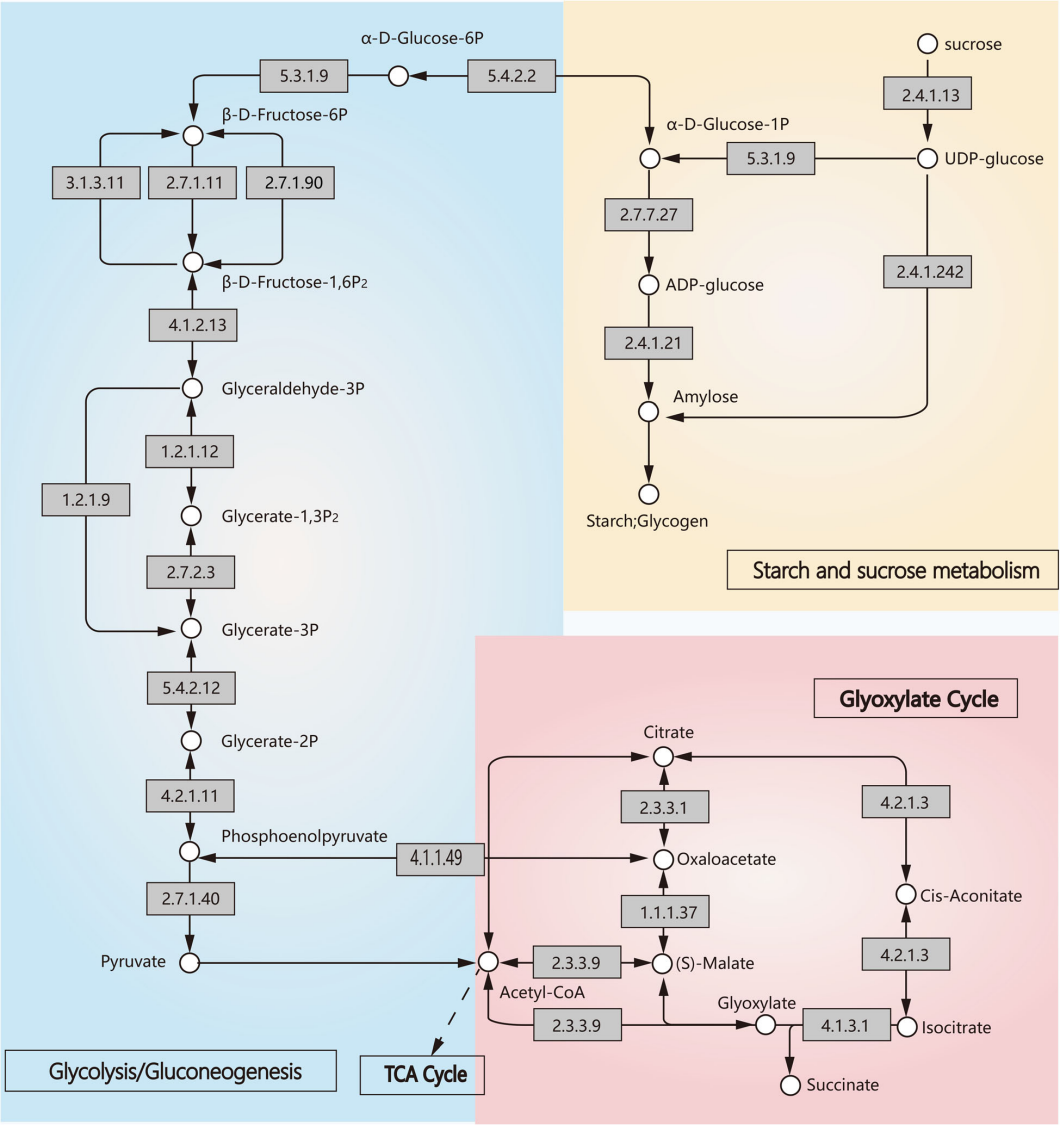
Network of starch and sugar synthesis in chestnut seeds

To identify the major metabolic pathways affecting chestnut seed development, KEGG pathway enrichment analysis of DEGs was performed. The ten most enriched KO terms of each group are listed in Table S2. DEG KO terms related to ribosomal and DNA replication were significantly enriched in the overall process of seed development. The four most enriched DEG KO terms in stage S3 were glycolysis, gluconeogenesis, fatty acid biosynthesis and oxidative phosphorylation. Considering that the expression of the genes related to starch and sucrose metabolism and gluconeogenesis differed significantly in different starch accumulation periods in chestnut seeds, a network of the starch and sugar synthesis pathways in chestnut seeds was constructed (Fig. 6). The network indicated that the starch synthesis pathway and glycolysis pathway shared the same precursor pool, including g-1-p and g-6-p.

The gene encoding ADP-glucose pyrophosphorylase (EC 2.7.7.27) was highly expressed during the peak period of starch accumulation (S3). The encoded protein catalyzes the conversion of g-1-p to adp-glucose, which is the direct precursor of starch synthesis. The expression of a gene encoding starch granule-bound starch synthase (EC 2.4.1.242), which is responsible for the synthesis of amylose and amylopectin, was also increased. Moreover, fructose diphosphate aldolase (FBA, EC 4.1.2.13), enolase (EC 4.2.1.11) and pyruvate phosphokinase (EC 2.7.9.1) were significantly regulated by protein phosphorylation. FBA and enolase catalyze reversible reactions in glycolysis. PPDK promotes gluconeogenesis by catalyzing the mutual conversion of pyruvate and PEP. The pyruvate and PEP production steps are initially irreversible in glycolysis. The above results indicated that glycolysis is inhibited while gluconeogenesis is enhanced, resulting in the flux of G-1-P and G-6-P into the starch synthesis pathway in chestnut seeds.

### Verification by qRT-PCR

**Fig. 7:**
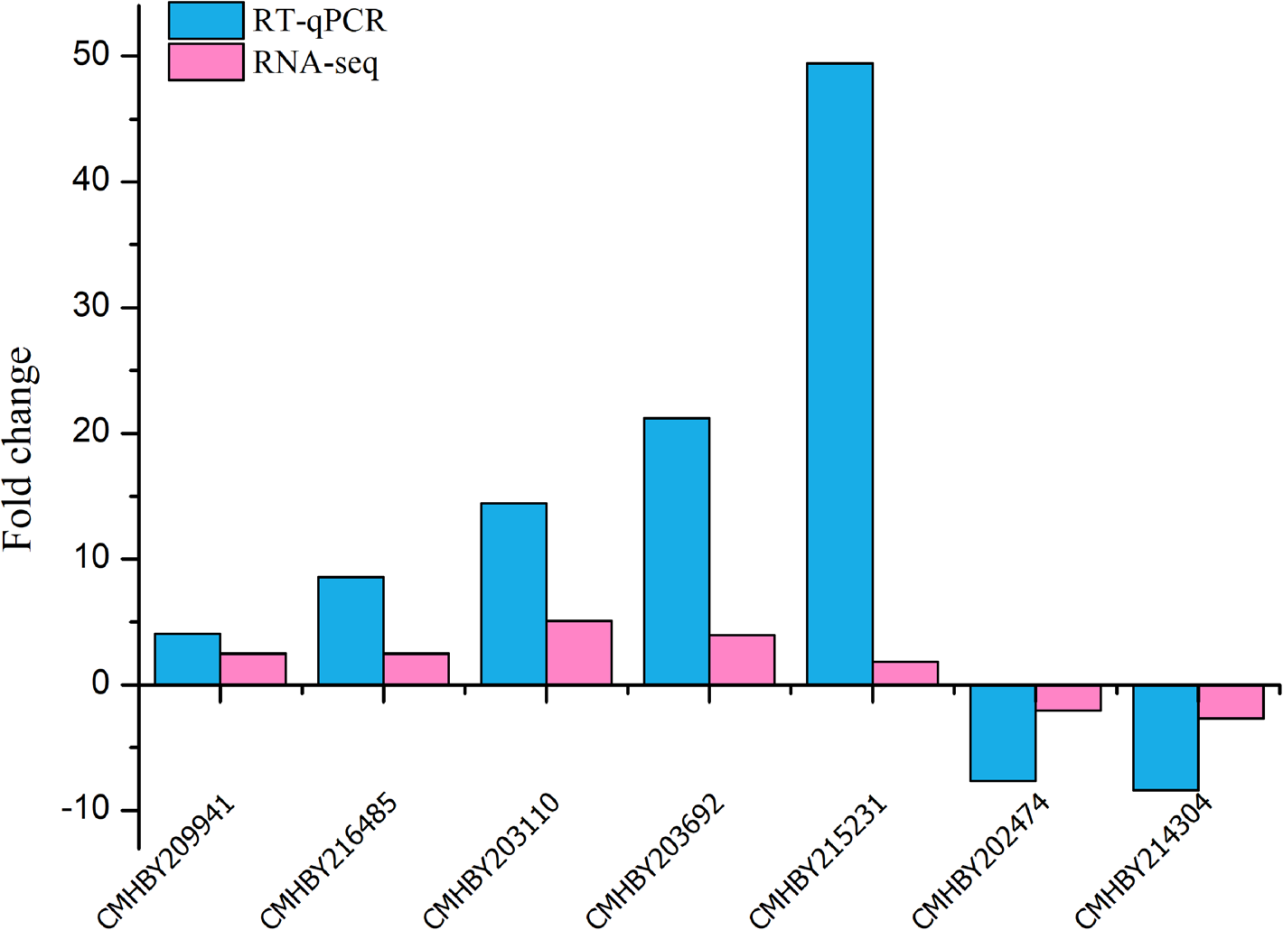
Expression of seven selected genes measured by RNA sequencing and qRT-PCR

To verify the reliability of the RNA sequencing data, qPCR was used to evaluate the expression profiles of five upregulated and two downregulated genes. The expression trends of the seven selected DEGs were consistent with the transcriptome data (Fig. 7), indicating that the two methods were reliable and complementary to each other for estimating gene expression.

## Discussion

The chestnut is a member of the Fagaceae family and is an economically important crop species. It has received extensive attention because of its high nutrient content and health-promoting properties [24]. Chestnut fruit development is accompanied by starch accumulation during the ripening process, in which sugar synthesis and decomposition pathways play a key role [21]. Studies have shown that different varieties of chestnut are characterized by different processes during development and maturation [19]. It has been demonstrated that the levels of sugar and starch are significantly higher under cross-pollination than self-pollination treatments during ovary development in chestnuts [25]. Our results are consistent with those of previous studies. In this study, the metaxenia effect was shown to lead to differences in the accumulation of starch in seeds produced from different pollination combinations in chestnuts, and cross-pollination resulted in a higher starch content than self-pollination. In addition, our research showed that metaxenia affects the size and color of nuts, which is consistent with findings in other species, such as hazelnuts [26] and tomatoes [27].

We coupled transcriptomic analysis with qPCR assays for selected genes to understand the global gene expression involved in starch and sucrose metabolism in three different type of Chinese chestnuts (Respectively YFF1, YFR1, YFM1). By analyzing the transcriptomes of three chestnut fruits at three different developmental stages, we found that sugar and starch content, SS and SSS activities all showed significant epigenetic effects during the ripening process of chestnut. In the early stage of seed development, energy metabolism, organic acid metabolism and translation processes are activated in large quantities, which promotes the synthesis of ATP, so that the seeds get more energy; and in the middle and late stages, accompanied by the activation of starch and sugar metabolism, promoted the accumulation of carbohydrates in chestnut.

Glycosyltransferases (GTFs) are enzymes (EC 2.4) that establish natural glycosidic linkages. They catalyze the transfer of saccharide moieties from an activated nucleotide sugar (also known as the “glycosyl donor”) to a nucleophilic glycosyl acceptor molecule, the nucleophile of which can be oxygen carbon, nitrogen, or sulfur based [28, 29]. In our study, the expression levels of genes encoding the glycosyl hydrolase family and phosphoglucomutase/phosphomannomutase were shown to play an important role in the seeds from all three pollination combinations. Glycosyl hydrolase controls the hydrolysis of glycosyl groups and participates in energy metabolism, and the change trend of this enzyme was opposite that of the accumulation of starch. The results showed that the most abundantly expressed unigenes according to total RPKM values at 45 DAF and 75 DAF included GBSS (EC 2.4.1.186). Studies on rice have shown that if the GBSS gene is mutated, deleted or transferred to the antisense GBSS gene, the mRNA amount, GBSS activity and amylose content of the GBSS gene will be significantly reduced. However, the transfer of the sense GBSS gene will increase the amount of starch accumulation [30]. The RPKM of GLG (EC 2.4.1.242) in the transcriptome was extremely low at 45 DFA and undetectable at 75 DAF. The GBSS and GLG gene families were identified from glycosyltransferase categories according to the KEGG protein database, which indicated that GTFs also play a very important role in the development of chestnut seeds [21]. However, another study indicated that the SuS (EC2.4.1.13) and SEB (EC 2.4.1.18) genes were actively expressed at 82 and 94 DAF and that SP (EC 2.4.1.1)-encoding unigenes were significantly downregulated at 94 DAF. GTFs seem to be less important in the process of seed development [19]. A gene encoding ADPase (EC 2.7.7.27) was shown to be highly expressed in the peak period of starch accumulation (94 DAP), which is consistent with the findings of Li et al. [19]. In other species, these are the main enzymes that affect starch accumulation from xenia. At 14 DAP in maize, the ADPase and neutral invertase (EC 3.2.1.26) activities were higher under cross fertilization than under self-fertilization. This advantage depended on the cross for the sucrose content, acid invertase activity, and SuS activity [13].

In addition to the enzymes closely related to sugar metabolism, the results of GO and KEGG enrichment analysis showed specificity in the seeds of different pollination combinations and in different developmental stages. A previous study showed that many metabolic pathways vary significantly during fruit development of the date palm, and carbohydrate metabolism (especially sugar synthesis) is particularly prominent during fruit ripening [31]. In stage S3 (94 DAP), amide metabolism, monocarboxylic acid metabolism, organic acid metabolism, and protein synthesis were significantly enriched. The seeds of the different pollination combinations were also characterized by many specific enriched terms. In YFF, GO terms such as carbohydrate derivative metabolic process, organophosphate biosynthetic process, organophosphate metabolic process, and cellular catabolic process were significantly enriched, and in YFR, DNA metabolic process and amino acid metabolic process were specifically enriched. To fully understand the biosynthesis of starch and its regulation, it will be very important to study these abundant pathways and related genes in the future. To more systematically analyze the differences in gene expression and regulatory networks during the development of chestnut kernels, we identified coexpressed gene sets by WGCNA. At 70 DAF, we identified six genes (Fig. 5a) at the core position, most of which were related to translation, cell proliferation, and sugar synthesis. At 82 DAF, a module with the highest correlation was identified, and this module exhibited transport-related activities. At this stage, both SS and sugar metabolism in chestnut kernels showed high activity, which might be one of the reasons for the high transport activity observed. In the last stage, we identified TFs related to plant growth, hormone regulation and biotic and abiotic stress responses, such as ACT and LIM TFs, which may be involved in the regulation of cell senescence and death in chestnut seeds.

## Methods

### Plant materials and sampling

The experimental site was located in Weidi Township, Yongren County (Lat. 25°51′ ~ 26°30′ N, 101°14′ ~ 101°49′ E, Alt. 1530 ~ 1700 m)), Yunnan Province. ‘Yongfeng 1’ (Yunnan Province certification local fine variety) was selected as the female parent. The following pollination combinations were established: ‘Yongfeng 1’ × ‘Yongfeng 1’ (YFF), ‘Yongfeng 1’ × ‘Yongren Zao’ (YFR), and ‘Yongfeng 1’ × ‘Yimen 1’ (YFM). Four sampling directions (east, west, north and south) were selected for performing the pollination combinations in the above groups. At the early stage of the germination of female flowers (2018/04/29), approximately 80 samples from each pollination combination were bagged. At the flowering stage of male flowers, fresh pollen was collected for direct pollination (2018/05/19) and was removed from the bag prior to thorn ball swelling (2018/05/26).

Description of chestnut varieties: ‘Yongfeng 1’ is a real-breeding variety in Yongren County, Yunnan Province, with an average single seed weight of approximately 16 g. ‘Yongren Zao’ is also a real-breeding variety in Yongren County, Yunnan Province, with an average single-grain weight of 12 g, in which the mature period occurs in late July. ‘Yimen 1’ is a well-approved variety in Yunnan Province. Its fruit ripening period occurs in mid-August, and it presents an average single-grain weight of 14.51 g.

Starting approximately 20 days before maturation, samples were collected every 11 days, and the last samples obtained consisted of mature fruits with thorn ball dehiscence. Chestnut seeds were collected from 18 pollination trees at three developmental stages (70, 82 and 94 DAP) (Fig. 1). The endosperm was removed from the seed and divided into three equal parts. Then, two of the parts were wrapped with silver paper and immediately stored in liquid nitrogen until being used for RNA extraction, and the third part was brought directly to the laboratory, where it was dried and ground.

### Physiological and biochemical indexes of sugar and starch

The seed endosperms were dried in a 60 °C oven. The soluble sugar content was determined via the colorimetric anthrone method [32]. The reducing sugar content was determined by direct titration [33], and the amylopectin and amylose contents were determined using a microanalytical method [34]. The SSS and SS levels were tested by double-antibody sandwich ELISA [35].

### Gene expression analyses

Total RNA from each seed sample was isolated using TRIzol reagent (Invitrogen, Carlsbad, CA, USA), and an Agilent 2100 system was used to detect RNA. Sequencing libraries were constructed by using the NEBNext® Ultra™ RNA Library Prep Kit for Illumina® (NEB, USA) according to the manufacturer’s recommendations. mRNA was enriched with mRNA capture beads and then fragmented. First-strand cDNA was synthesized using random hexamers, followed by synthesis of double-stranded cDNA. After end repair, an A tail and an adapter were added, and cDNA fragments 150–200 bp in length were selected. Sequencing libraries were generated via PCR. Then, we measured the quality of the libraries. The libraries were sequenced using a HiSeq 2000 sequencer (Illumina, San Diego, CA, USA), and 150 bp paired-end reads were generated. Adapters and low-quality reads were removed from the raw data to obtain clean data. We also measured the Q20 and Q30 values of the clean data. The high-quality cleaned reads were mapped to the Chinese chestnut reference genome [22] using HISAT 2 with default parameters. The unique mapping read counts were normalized to FPKM values by Cufflinks (v2.0.2) to obtain the relative expression levels for each sample.

PCA was performed to show the correlations between biological replicates using an R package. We performed differential expression analysis based on Cufflinks. Genes with adjusted *P*-values (q value) ≤ 0.05 and an absolute log2 (fold change) value were identified as DEGs. To observe the gene expression patterns in seeds in different stages, the DEGs were grouped by hierarchical clustering according to their level of expression using the pheatmap R package.

### Functional enrichment analysis

We performed GO enrichment analysis of the DEGs according to GO:TermFinder [36]. Significantly enriched GO terms for DEGs compared with the genomic background were identified using a hypergeometric test. A corrected *p* value < 0.05 was set as the threshold for significantly enriched GO terms. Similarly, we identified the statistical enrichment of DEGs in KEGG pathways using KOBAS software (v2.0). MapMan (v.3.5.1R2) was used to visualize the expression patterns of DEGs.

### Weighted gene coexpression network analysis

To detect patterns of gene connectivity, we analyzed the data via WGCNA with the WGCNA package (v1.51) in R. Genes with a low coefficient of variation of the averaged RPM (CV < 1) among all samples were discarded, and the remaining 12,662 genes were used for the analysis.

First, the scale-free gene network condition should be satisfied before conducting WGCNA. Moreover, it is necessary to define the correlation matrix of gene coexpression and adjacency function. A soft threshold (power) of five was chosen in this study. Then, the dissimilarity measurements of different nodes were calculated, and a hierarchical clustering tree was built based on these data. Different dendrogram branches represent various modules. We drew a heatmap via the module eigengene that was used to show the expression pattern in every sample. Therefore, we can study a module that is significantly related to further research via heatmap data. The functional significance of the modules was evaluated through GO and KEGG enrichment analysis according to a previous method. The coexpression networks of selected modules of genes were visualized by Cytoscape (version 3.1.0).

### qRT-PCR identification

The expression patterns of 7 unigenes encoding SSS and SS in developing Chinese chestnut seeds were studied by qRT-PCR. Total RNA was extracted using a Qiagen RNeasy Mini Kit (Qiagen Inc., Valencia, CA) and was reverse transcribed into cDNA by using random primers. The internal control was 18S rRNA [37]. The 2(−△△CT) method was used to analyze the data [38].

## Supplementary Information

**Additional file 1: Figure S1.** Pearson correlation between samples. **Figure S2.** DEGs and GO annotations in different stages of seeds from three pollination combinations. A: DEGs in different stages. B: GO annotations in different stages. **Figure S3.** WGCNA of all KEGG pathways. A: Hierarchical clustering of unigenes and module identification. B: Relationships between gene modules and sample groups. **Table S1.** Statistical analysis of transcriptome sequencing in seeds from three pollination combinations. **Table S2.** The ten most enriched KO terms of each group are listed.

## Data Availability

Sequencing data are available via the NCBI Bioproject PRJNA540079.

## Abbreviations

DEGs: Differentially expressed genes
FPKM: Fragments per kilobase of transcripts per million mapped fragments
