# Supplementary figures and images for: Transcriptome sequencing and differential expression analysis of seed starch accumulation in Chinese chestnut Metaxenia
Source: BMC Genomics. 2021 Aug 13;22:617. doi: 10.1186/s12864-021-07923-5 (PMC8362260; doi:10.1186/s12864-021-07923-5)

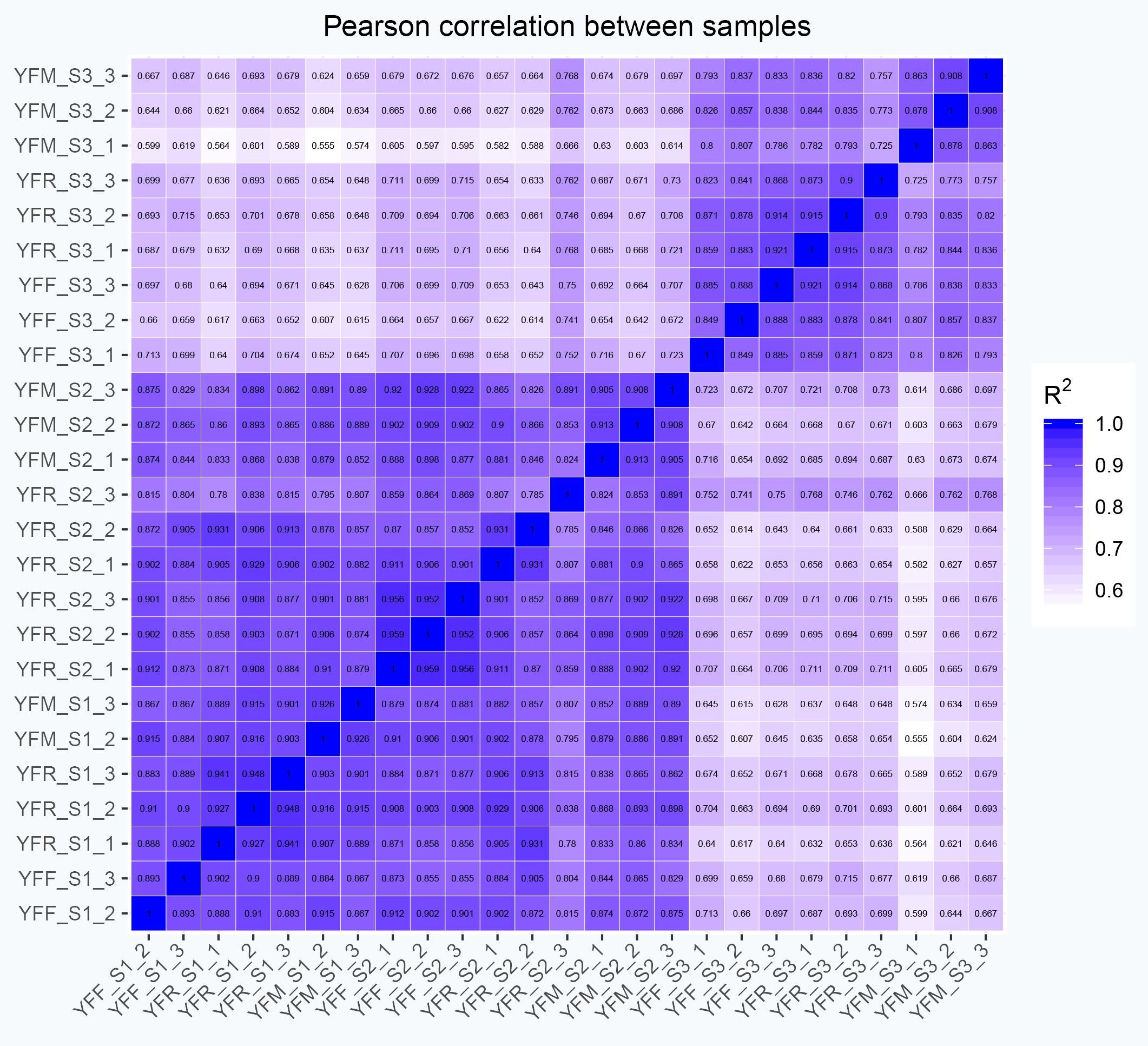

Supplement: Supplementary file 1 — Additional file 1: Figure S1. Pearson correlation between samples. Figure S2. DEGs and GO annotations in different stages of seeds from three pollination combinations. A: DEGs in different stages. B: GO annotations in different stages. Figure S3. WGCNA of all KEGG pathways. A: Hierarchical clustering of unigenes and module identification. B: Relationships between gene modules and sample groups. Table S1. Statistical analysis of transcriptome sequencing in seeds from three pollination combinations. Table S2. The ten most enriched KO terms of each group are listed. [file 12864_2021_7923_MOESM1_ESM.zip › fig S1.tif]

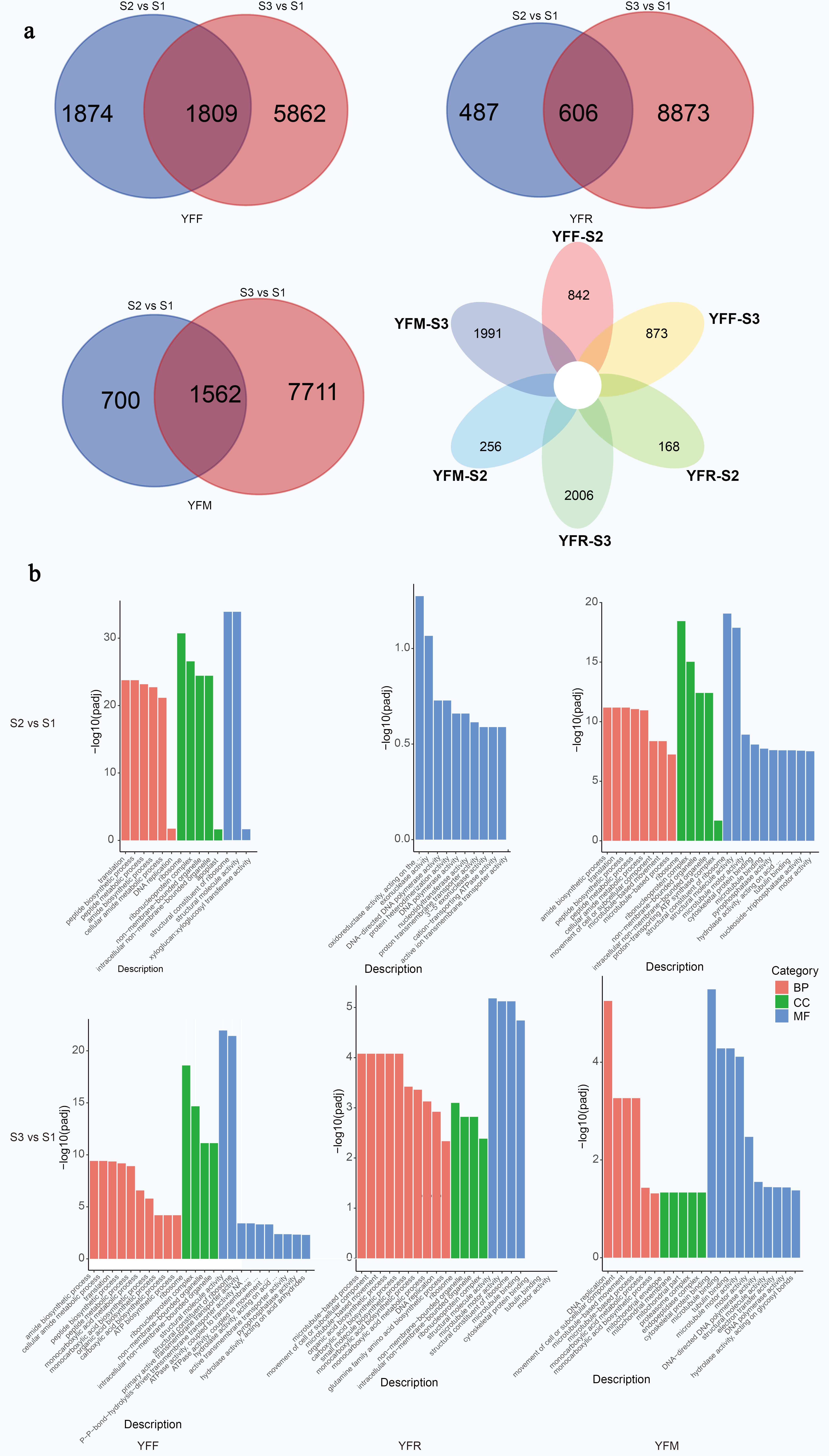

Supplement: Supplementary file 1 — Additional file 1: Figure S1. Pearson correlation between samples. Figure S2. DEGs and GO annotations in different stages of seeds from three pollination combinations. A: DEGs in different stages. B: GO annotations in different stages. Figure S3. WGCNA of all KEGG pathways. A: Hierarchical clustering of unigenes and module identification. B: Relationships between gene modules and sample groups. Table S1. Statistical analysis of transcriptome sequencing in seeds from three pollination combinations. Table S2. The ten most enriched KO terms of each group are listed. [file 12864_2021_7923_MOESM1_ESM.zip › fig S2.tif]

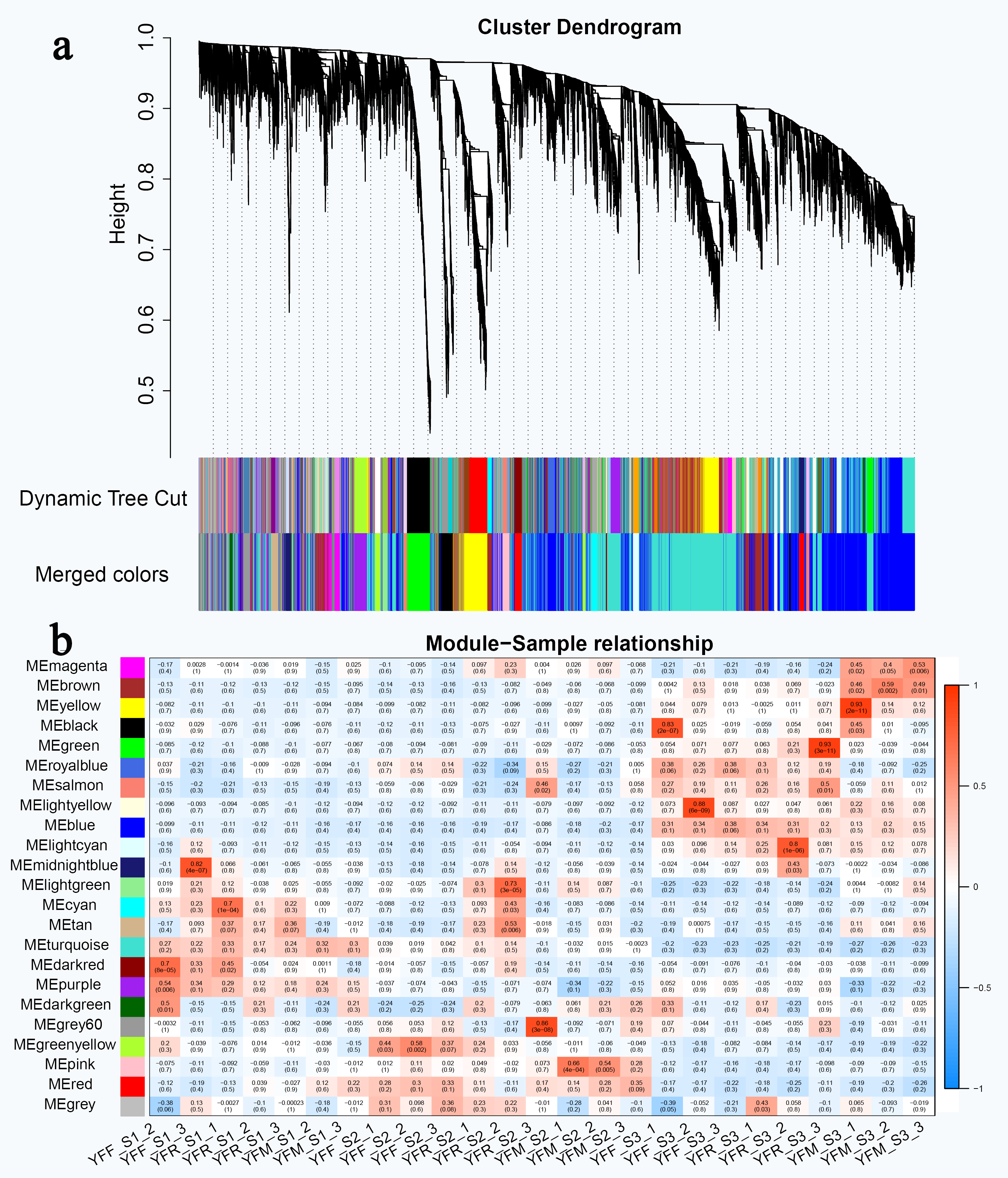

Supplement: Supplementary file 1 — Additional file 1: Figure S1. Pearson correlation between samples. Figure S2. DEGs and GO annotations in different stages of seeds from three pollination combinations. A: DEGs in different stages. B: GO annotations in different stages. Figure S3. WGCNA of all KEGG pathways. A: Hierarchical clustering of unigenes and module identification. B: Relationships between gene modules and sample groups. Table S1. Statistical analysis of transcriptome sequencing in seeds from three pollination combinations. Table S2. The ten most enriched KO terms of each group are listed. [file 12864_2021_7923_MOESM1_ESM.zip › fig S3.tif]
